# New Appliances and Things Medical

**Published:** 1907-04-06

**Authors:** 


### NEW APPLIANCES AND THINGS MEDICAL.

[We shall be glad to receive at our Office, 28 & 29 Southampton Street, Strand, London, W.C., from the manufacturers, specimens of all new preparations and appliances which may be brought out from time to time.]

### MIOL.

(Miol Manufacturing Co., 68 Southwark Bridge Road, S.E.)

A Specimen of this recently prepared substance has been submitted to us. It consists essentially of malt extract, oil (Provence Cream Oil), iodine, phosphorus, and cinamic acid. It is recommended in pulmonary tuberculosis as a substitute for cod-liver oil. Its nutritive value is stated to be equal, if not superior, to the former preparation, and it contains, in addition, the medicinal substances mentioned above. Of these great importance is to be attached to the iodine, which appears to exert, upon the pathological processes taking place in the lung, the favourable influence of potassium iodide without producing any of the depressant or otherwise undesirable effects of this remedy. Cinamic acid as such, as is probably known to our readers, has received extensive employment in phthisis, and seems to exert a beneficial influence. The phosphorus stimulates nutrition by increasing the assimilation of proteids and fats. Miol does not possess an unpleasant taste, and seems from *à priori*, as well as from *à posteriori*, reasons well worthy of an extensive trial.

### OKOL.

(The Sanitas Company, Limited, Locksley Street, Limehouse, London, E.)

The Sanitas Company have recently prepared a new disinfectant, to which the name Okol has been given. This substance is a carefully prepared emulsion, and its germicidal properties have been carefully examined by Dr. Wynter Blyth. Since the Sanitas Company first prepared Okol certain improvements have been effected in its manufacture, specially so far as concerns the emulsification of the disinfectant principle. The remarks which follow refer to the so-called "New Okol." It has been found by bacteriological research that exposure of a 24 hours old culture in nutrient broth of typhoid bacilli for 2½ minutes to new Okol in a dilution corresponding to 1 in 1,900 renders them incapable of further growth. This substance thus possesses a very strong germicidal power; in fact, when compared with phenol its germicidal value is 22. We think the preparation will recommend itself to the profession, and it seems to be in the dilution required an exceedingly bland product, and the rigorous bacteriological test which it has undergone has placed its reliability beyond dispute.

